# Phosphorylated ERK is a potential prognostic biomarker for Sorafenib response in hepatocellular carcinoma

**DOI:** 10.1002/cam4.1228

**Published:** 2017-10-13

**Authors:** Yuelong Liang, Jiang Chen, Qingsong Yu, Tong Ji, Bin Zhang, Junjie Xu, Yi Dai, Yangyang Xie, Hui Lin, Xiao Liang, Xiujun Cai

**Affiliations:** ^1^ Department of General Surgery Sir Run Run Shaw Hospital College of Medicine Zhejiang University Hangzhou Zhejiang 310016 China

**Keywords:** Biomarkers, Phosphorylated ERK, Sorafenib, Hepatocellular carcinoma

## Abstract

Sorafenib, the only approved drug for hepatocellular carcinoma, acts as a remarkable inhibitor of Raf serine‐threonine kinases. However, Sorafenib is expensive, and clinical experience shows that it is not an effective treatment for many patients. Previous study has demonstrated that phosphorylated ERK (pERK) is a key downstream component in the RAF/MEK/ERK signaling pathway. Here, we investigate whether pERK is a useful biomarker for treating HCC with Sorafenib. In vitro cell viability assays showed that the efficacy of Sorafenib was distinctly different according to the level of pERK. Furthermore, in established patient‐derived xenografts from HCC specimens, we found that the growth rate of tumors with high levels of pERK was significantly decreased by Sorafenib treatment. Taken together, pERK is a potential biomarker for the sensitivity to Sorafenib in treating HCC.

## Introduction

Hepatocellular carcinoma (HCC) is one of the most common types of cancer worldwide. Due to its high malignancy, HCC is the third leading cause of cancer‐related mortality worldwide 1, and in China, it ranks second. An epidemiological statistic published in the *New English Journal of Medicine* stated that a great majority of HCC patients experience relapse, with a 5‐year survival rate not more than 10% 2. Unfortunately, there are no new treatments that can improve survival in patients with advanced hepatocellular carcinoma, and HCC is not sensitive to either chemotherapy or radiotherapy 3, 4.

The development of targeted therapies for cancers, such as trastuzumab (against HER2‐positive breast cancer) 5, 6, tamoxifen (against estrogen receptor‐positive breast cancer) 7 and cetuximab (against EGFR‐positive non‐small‐cell lung carcinoma) 8, has a strong positive response on improving disease‐free survival and overall survival for cancer patients. Therefore, new targeted therapies for HCC are both promising and urgently needed.

As a multi‐kinase inhibitor, Sorafenib inhibits Raf serine/threonine kinase and afterward blocks the Raf/MEK/ERK pathway. Sorafenib is the only molecularly targeted agent that has been confirmed as efficacious in treating advanced HCC. In phase III and phase II trials, Sorafenib exhibits significant survival benefits for patients with advanced HCC 9, 10. Even so, the efficacy of Sorafenib in treatment of HCC remains moderate and patients survival is short following Sorafenib treatment 11. Sorafenib therapy is both expensive 12 and unsatisfactory 13. There is an urgent need for a powerful new biomarker to predict sensitivity to Sorafenib in HCC therapy.

In this research, the relationship between expression levels of phosphorylated ERK, a well known key downstream factor in the RAF/MEK/ERK signaling pathway, and Sorafenib response was examined using cell lines and patient‐derived primary HCC xenografts in a mouse model. The results demonstrated that HCC characterized by higher levels of pERK are more sensitive to Sorafenib. Our study indicates that pERK levels may be used to predict the efficacy of Sorafenib in treating HCC.

## Materials and Methods

### Chemicals and other reagents

Sorafenib was weighed and stored in dry form away from light. For in vitro experiments, the Sorafenib was dissolved in DMSO, and the concentration of DMSO was kept under 0.1%. For in vivo experiments, Sorafenib was dissolved in 50% cremophor EL (Sigma, St Louis, Mo) and 50% ethanol. The compounds were sonicated for 5–10 min. The aqueous mixture (75% water) was gradually added and the final dosing solution was generated 14. 30 mg/kg of the above mixture was administered daily by oral gavage.

### Cell culture

HepG2 cell line was obtained from ATCC. Primary cell HCC‐0010 was gained from Patient‐derived xenografts (by Professor Cang Yong). All cells were grown in appropriate medium containing 10% fetal bovine serum. Cells were cultured in a humidified 37°C incubator with an atmosphere of 5% CO_2_. CCK‐8 (Dojindo, Kumamoto, Japan) was used to test the proliferative potential of HCC cells.

### Patient samples

Use of human tissues was approved by the Sir Run Run Shaw Hospital ethical committee. Written informed consent was obtained from each patient. Specimens of the patients were collected from those undergoing liver resection for HCC at the Sir Run Run Shaw Hospital (Zhejiang University, Hangzhou, China).

### Patient‐derived primary HCC xenografts model

Patient‐derived xenografts were implanted subcutaneously into nude mice (female, age 6–8 weeks). Tumor growth was monitored, and dimensions of xenografts were measured after 1 month of Sorafenib treatment. The following formula was used to calculate tumor volume: (longest tumor diameter)* (shortest tumor diameter)^2^/2.

All mice were maintained according to the Guide for the Care and Use of Laboratory Animals published by the NIH. Sterilized food and water ad libitum were provided. All mice were housed in negative pressure isolators with 12 h light/dark cycles.

### Western blot

For whole protein extracts, cell samples, and tumor tissues were homogenized in Rapabuffer (sigma) with a protease inhibitor cocktail (Roche). The mixture was then incubated on ice for 30 min. Soon afterwards, the compounds were centrifuged for 15 min at 12,000*g*. 4–12% SDS–PAGE gel and nitrocellulose membrane (Invitrogen) were used to separate protein. The membranes, with the primary antibodies, were incubated overnight at 4°C. Immunoblots were developed using secondary antibodies such as anti‐mouse‐ or anti‐rabbit‐HRP (SANTA CRUZ). Enhanced chemiluminescence (Pierce Biotechnology) was performed according to the instructions. The antibodies used for Western blotting included Phospho‐p44/42 MAPK (Erk1/2) (Thr202/Tyr204) (Cell Signaling Technology, 1:2000), p44/42 MAPK (Erk1/2) (Cell Signaling Technology, 1:2000), *β*‐actin (Cell Signaling Technology, 1:2000), SETD7 (Cell Signaling Technology, 1:1000).

### Immunohistochemistry

Paraffin embedding was used to process specimens. Sections of 5 *μ*m were prepared for subsequent experiments. Endogenous peroxidase activity and non‐specific staining were blocked, then the sections were incubated overnight with primary antibodies at 4°C. The strept avidin‐biotin peroxidase complex method was used according to the instructions (Lab Vision). The antibodies used for immunohistochemistry included Phospho‐p44/42 MAPK (Erk1/2) (Thr202/Tyr204) (Cell Signaling Technology, 1:200).

### Statistical analysis

Mean ± SD were used to represent experimental data. An unpaired Student's *t*‐test or Mann–Whitney rank sum test was used to compare the two groups. The *χ* ^2^‐test was used to test the differences in mortality. *P *<* *0.05 was considered as significant.

## Results

### The efficacy of Sorafenib in HCC was different between different levels of pERK in HepG2 cells and HCC‐0010 cells

**Figure 1:**
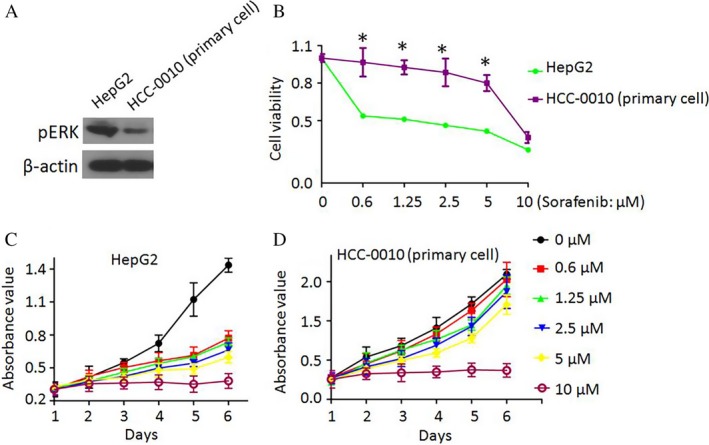
Effects of Sorafenib on cell proliferation differ between HepG2 cells and HCC‐0010 cells. (A) Basal pERK levels in different cell lines were measured by western blotting. (B–D) The effects of Sorafenib on cell proliferation were measured by the CCK‐8 cell viability assay (mean±SEM; *P<0.05).

The western blot comparison shows that the Chinese origin cells (HCC‐0010) contain significantly lower levels of pERK than HepG2 (Fig. 1A). The CCK‐8 cell viability assay, used to measure the efficacy of Sorafenib against HCC showed that different HCC cell lines displayed distinct responses upon Sorafenib treatment (Fig. 1C). For instance, Sorafenib strikingly inhibited HepG2 cell proliferation in a dose‐dependent manner, while HCC‐0010 cells showed less sensitivity to Sorafenib‐mediated proliferation inhibition (Fig. 1B).

### Basal pERK levels vary in HCC patients

**Figure 2:**
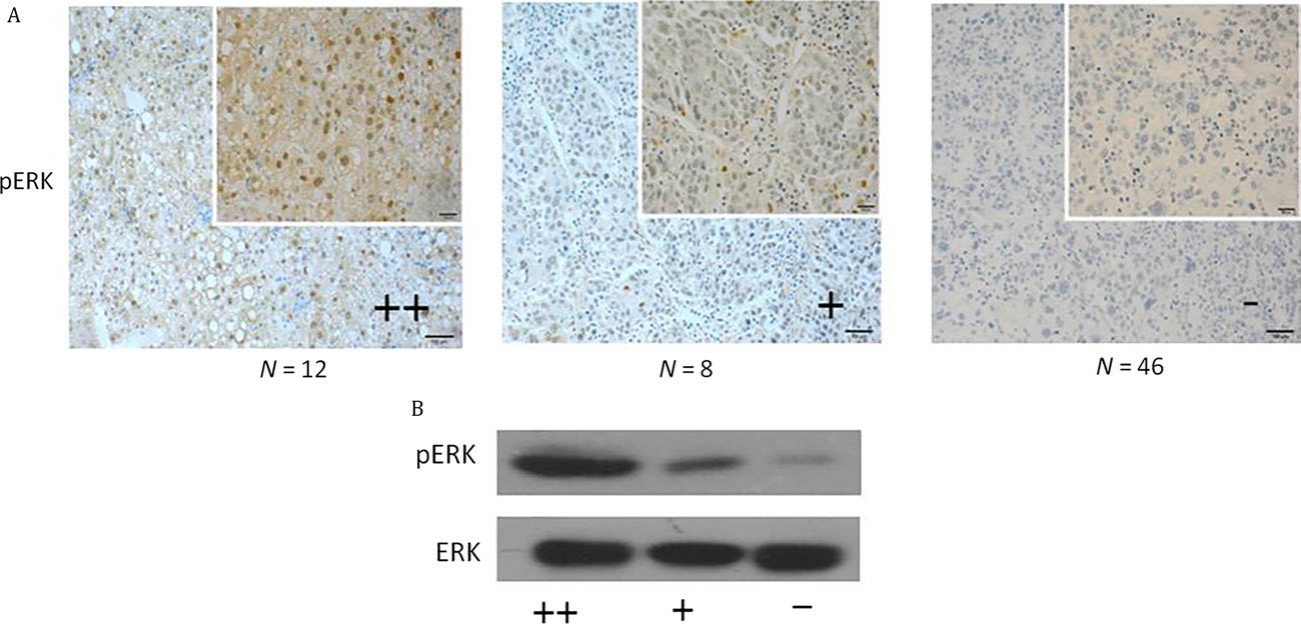
Different levels of pERK in HCC patient samples were observed. Basal pERK levels in different HCC patients were measured by immunohistochemistry (A) and western blotting (B).

*pERK* is one of the most important key factors of the RAf/MEK/ERK pathway. To determine whether there is any relationship between basal pERK levels and the efficacy of Sorafenib on killing cells in vivo, pERK levels in different HCC patients were compared by immunohistochemistry (Fig. 2A) and western blot (Fig. 2B). The pERK expression levels were examined by immunohistochemistry in 66 HCC patient samples and only 20 patients (30.3%) showed high levels of expression.

### Effect of Sorafenib on tumor growth is correlated with basal pERK levels in patient‐derived primary HCC xenograft models

**Figure 3:**
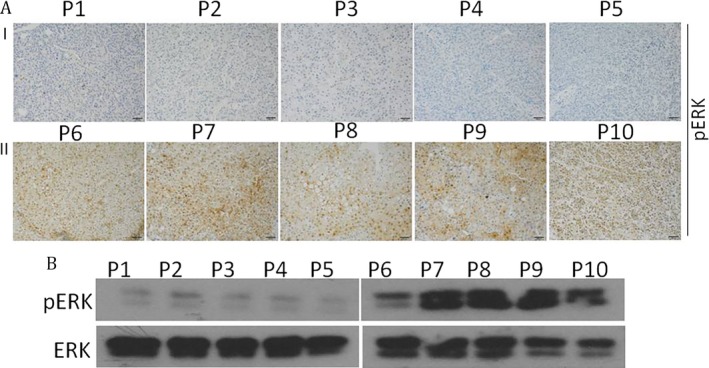
Patient‐derived primary HCC xenografts models with different expression of pERK were established. Expression of pERK in patient‐derived primary HCC xenograft models was verified by western blotting (B) and Immunohistochemistry (A).

**Figure 4:**
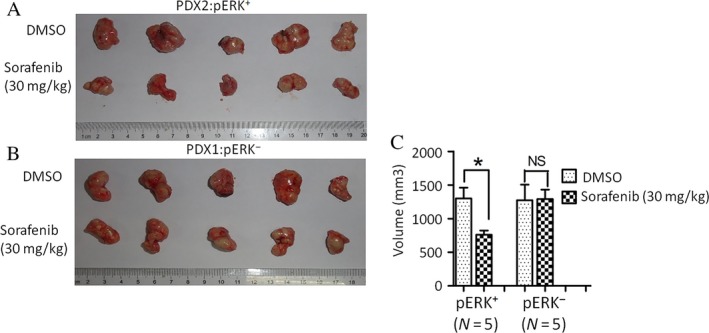
Effects of Sorafenib on tumor growth were significantly correlated with basal pERK levels in patient‐derived primary HCC xenograft model. (A and B) The effects of Sorafenib in patient‐derived primary HCC xenograft models with different expression of pERK were evaluated. (C) The volume of patient‐derived primary HCC xenograft models treated with 30 mg/kg Sorafenib or DMSO (mean±SEM; *P<0.05).

To further verify the relationship between pERK level and efficacy of Sorafenib, we selected five pairs of patient‐derived primary HCC xenograft models according to the expression levels of pERK: I and II (high levels of pERK, No. 1‐5; low levels of pERK, No. 6‐10) to evaluate the effects of Sorafenib in vivo (Fig. 3A and B). Mice in these two groups were treated with either DMSO or 30 mg/kg Sorafenib daily by gavage and tumor sizes were measured after 1 month. The growth rate of tumors in group I mice had obviously decreased with the Sorafenib treatment while Sorafenib treatment had little effect on tumor growth in group II mice (Fig. 4A and B). Tumors in Sorafenib‐treated mice had shrunk obviously in Group I than in Group II, compared with the DMSO‐treated controls (Fig. 4C).

### Gene expression was different between HepG2 cells and HCC‐0010 cells

**Figure 5:**
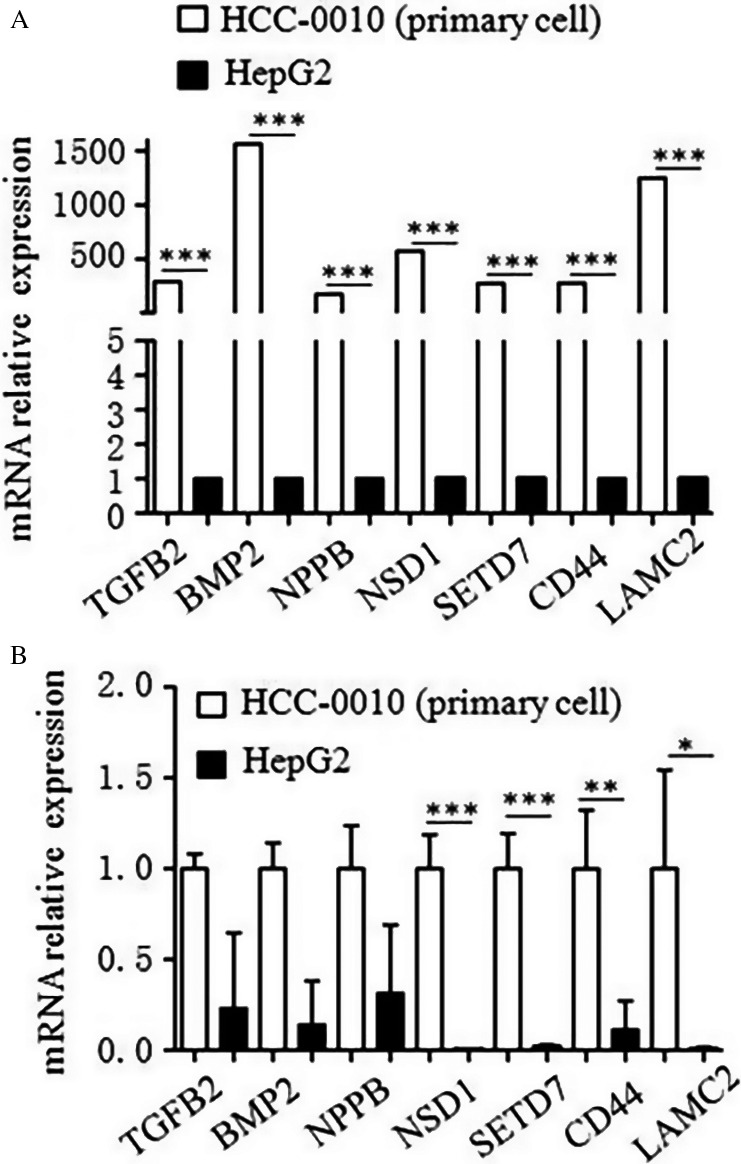
Gene expression was different between HepG2 cells and HCC‐0010 cells by RNA‐seq. (A) RNA‐seq was used to discover gene expression changes with Sorafenib treatment. (B) Real‐time qPCR was used to verify the results of RNA‐seq in cell lines (mean±SEM; *P<0.05; **P<0.01; ***P<0.001).

To explore the underlying mechanisms, we carried out RNA‐seq to discover gene expression changes with Sorafenib treatment. Either DMSO or 2 *μ*mol/L Sorafenib were used to treat HepG2 and HCC‐0010 cells for 24 h and RNA‐seq was performed. We found that there were significantly more genes whose expression levels changed over fourfold after Sorafenib treatment in HepG2 cells than in HCC‐0010 cells (Table 1). More interestingly, we also observed genes involved in several important tumor‐associated pathways including the ALK pathway 15, 16, Lysine degradation 17 and ECM‐receptor interaction 18 showed dramatically higher levels of expression in HCC‐0010 cells compared with HepG2 cells (Fig. 5A, Table 2). This differential expression might explain the difference in sensitivity to Sorafenib between HCC‐0010 and HepG2 cell lines. Real‐time qPCR was used to verify the results of RNA‐seq in cell lines, which showed that Lysine degradation associated genes (NSD1 and SETD7) might be the key factors (Fig. 5B).

**Table 1:** The number of downstream genes regulated by Sorafenib

	The direction of change	The number of change^a^	For example
HepG2 (DMSO: Sorafenib)	Up	20	PGLYRP2; SLC6A12; ANXA9…
	Down	74	DUSP5; IGFBP1; FOS…
HCC‐0010 (DMSO: Sorafenib)	Up	11	NLRP14; EPHA7; COX4I2…
	Down	9	ICAM5; SHISA8: CD40LG…

a Changes of gene expression over four folds.

**Table 2:** Genes with high expression in HCC‐0010 cell lines

Gene	Chromosomelocation	Associated pathway	Fold_change^a^ (HCC‐0010/HepG2)	*P* value
TGFB2	chr1	ALK_PATHWAY	295	2.51E‐05
BMP2	chr20		1564	3.38E‐13
NPPB	chr1		175	8.50E‐08
NSD1	chr5	Lysinedegradation	576	1.03E‐05
SETD7	chr4		276	7.77E‐09
CD44	chr11	ECM‐receptorinteraction	280	2.72E‐05
LAMC2	chr1		1250	2.27E‐07

a Changes of gene expression over 150‐fold.

### Down‐regulation of SETD7 increased the sensitivity of HCC to Sorafenib

**Figure 6:**
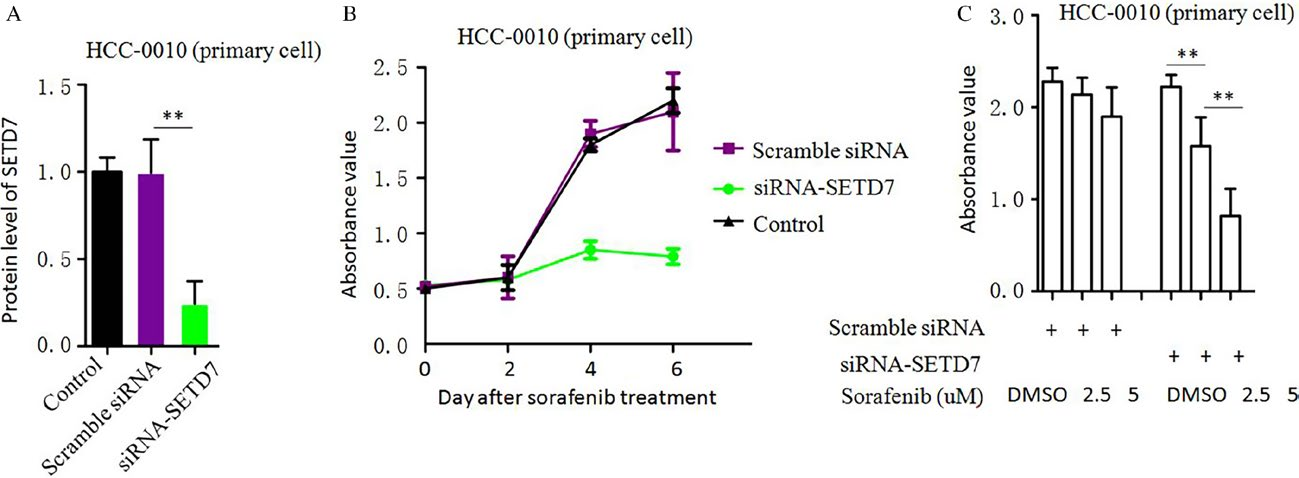
Down‐regulation of SETD7 increased the sensitivity of HCC to Sorafenib. (A) SETD7 protein expression was reduced by siRNA‐SETD7. (B, C) The effects of Sorafenib on cell proliferation were measured by the CCK‐8 cell viability assay (mean±SEM; **P<0.01).

Down‐regulation of SETD7 by siRNA‐SETD7 reduced the levels of protein expression compared with the Control/Scramble siRNA treated in HCC‐0010 cells (Fig. 6A). Together, results shown in Figures 6B and C suggest that Sorafenib alone has little effect on HCC‐0010, but the addition of siRNA‐SETD7 enhances the efficacy of Sorafenib to suppress HCC cell proliferation. These results suggest that SETD7 plays an important role in the pERK^−^ HCC that are resistant to Sorafenib.

## Discussion

The Ras/Raf/MEK/ERK signaling cascade is involved in proliferation, transformation, mitogenesis and differentiation of cancer cells, and is frequently deregulated in tumorigenic diseases 19, 20, 21, 22. The RAF/MEK/ERK cascade plays a major role in the pathogenesis of HCC, and many inhibitors against this cascade, including small molecular inhibitors and monoclonal antibodies, have been developed to treat HCC 23. Sorafenib (Nexavar, Bayer Health Care Pharmaceuticals) inhibits the Raf serine‐threonine kinases and blocks the RAf/MEK/ERK signaling pathway 24. It is the only known molecularly targeted agent approved for treatment of advanced HCC. However, clinical experience shows that Sorafenib is expensive and not all patients with HCC can obtain benefits from it. It was the urgent need to identify biomarkers that can predict the efficacy of Sorafenib against HCC. Biomarkers that could predict the likely benefits of Sorafenib in a specific patient would greatly help doctors make clinical decisions 25.

The pERK protein is a key downstream factor of the MEK/ERK cascade signaling pathway. Although this is an intuitive potential biomarker candidate, the present evidence (either in vitro or in vivo) to support using pERK as a predictive marker for Sorafenib in HCC therapy is preliminary. Zhang et al. evaluated the effects of Sorafenib on cell proliferation by cell viability assays in SMMC‐7721, MHCC97‐L, MHCC97‐H and HCCLM6, and thought that basal pERK expression levels were an important factor in Sorafenib efficacy. But in vivo experiments were lacking. We found previously that anti‐PD‐1 immunotherapy might complement Sorafenib in treating HCC patients. We had also identified that pERK would help HCC patient selection to achieve optimal clinical benefits. To examine the efficacy of Sorafenib on individual tumors, we generated patient‐derived xenografts using surgically removed HCC tissue. Unfortunately, we could only recruit three pairs of patient‐derived primary HCC xenografts at that time, and our results may have suffered from selection bias 26.

In this study, we assessed the activity of the RAF/MEK/ERK pathway in different HCC cell lines and HCC tissues from patients. Both Immunohistochemistry and Western blotting were used to determine the intracellular expression of pERK. In cell killing assays, Sorafenib inhibited proliferation of cancer cells with different pERK expression in a dose‐dependent manner. That the efficacy of Sorafenib is correlated with basal intracellular pERK levels was shown by correlation analysis between the IC50 values and the pERK density values, indicating that Sorafenib efficacy was closely related to the activity of the RAF/MEK/ERK signaling pathway in HCC tumor cells. This is consistent with the recent findings of the Zhang et al. in vitro study and our previous study 26, 27.To explore the underlying mechanisms, we carried out RNA‐seq to discover gene expression changes with Sorafenib treatment in HCC cell lines that differentially express pERK (HepG2 and HCC‐0010). We found out that there were significantly more genes whose expression levels changed over fourfold after Sorafenib treatment in the HepG2 cells relative to HCC‐0010 cells (Table 1). More interestingly, we also observed genes involved in several important tumor‐associated pathways including the *ALK* pathway (*TGFβ2*,* BMP2*,* NPPB and others*) 15, 16, Lysine degradation (*NSD1, SETD7 and others*) 17 and ECM‐receptor interaction (*CD44, LAMC2 and others*) 18 showed dramatically higher levels of expression in HCC‐0010 cells compared with HepG2 cells (Table 2). This might explain the different sensitivity of Sorafenib between HCC‐0010 and HepG2 cell lines. Sun et al. reported that HCC cells that are characterized by active epithelial‐mesenchymal transition (EMT) exhibit increased drug resistance, whereas SB‐431542, an antagonist of activin receptor‐like kinase (*ALK*) receptors, attenuates EMT in HCC cells 28. Huang et al. report that the activity of lysine‐specific histone demethylase 1A is required for the emergence of cancer cells following Sorafenib treatment, suggesting that KDM1A inhibitors may be utilized to alleviate acquired resistance to Sorafenib, thus increasing the therapeutic efficacy of Sorafenib in HCC patients 29. Fernando et al. described that a mesenchymal profile and expression of *CD44*, linked to activation of the TGF‐*β* pathway, may predict the poor response to Sorafenib in HCC patients 30. Govaere et al. identified a prominent role for laminin‐332, encoded by *LAMC2*, as part of the specialized CSC niche in maintaining and supporting cell stemness, which leads to chemo‐resistance 31. We used real‐time qPCR to verify the results of RNA‐seq in human tissues, and showed that Lysine degradation associated genes (*NSD1* and *SETD7*) were expressed at dramatically higher levels in HCC‐0010 cells than in HepG2 cells. Down regulation of *SETD7* increased the sensitivity of HCC to Sorafenib, indicating that *SETD7* may the key factor of Sorafenib resistance in HCC. As a methyltransferase for H3K4, *SETD7* (also known as *SET7, SET9, or SET7/9*) belongs to the SET domain‐containing proteins. SETD7 plays an important role in inflammation and oncogenesis. However, the functions and mechanisms of SETD7 in HCC remain poorly understood. Chen et al. reported that *SETD7* overexpression can promote HepG2 cell proliferation, whereas *SETD7* knockdown can inhibit SMMC‐7721 cell proliferation by regulating the cell cycle, indicating that *SETD7* plays a critical role in HCC [32]. The immunohistochemistry signature of *SETD7* may provide a clinical tool for personalized therapy of HCC patients treated with Sorafenib.

Only 30.3% (20 out of 66) patient samples from Chinese ethnicity showed high pERK expression. RNA‐seq data suggested that different HCC cell lines (HCC‐0010 (Chinese origin cells)) and HepG2 (Caucasian origin cells)) involve different tumor‐associated pathways, which partly explain the ethnic differences in Sorafenib efficacy. In our opinion, the pERK signal pathway is not a key tumor‐associated pathway in some Chinese HCC patients, and these patients will not benefit from taking Sorafenib.

To support our hypothesis in vivo, we established patient‐derived xenografts on mice. Preliminary studies demonstrated that the mouse model exhibits tissue and cellular characteristics that resemble human tumors 33, 34. Our investigations revealed that the growth rates of tumors with high levels of pERK expression in patient‐derived xenografts were significantly decreased by Sorafenib treatment, while the tumor with low or no expression of pERK did not react to Sorafenib. We previously reported results from 3 pairs of patient‐derived xeonografts 26. Here, we recruited another 5 pairs of xenografts, originated from 10 different patients for further in vivo validation. Unfortunately, we are still limited by the availability of different patient‐derived xeonografts.

In summary, this research further supports, in vitro and in vivo*,* that pERK could be a useful biomarker to predict the actions of Sorafenib, and partly explains the ethnic differences in its efficacy, providing a basis for personalized medicine.

## Conflict of Interest

We declare that no competing of interest and conflicts of ethics involved in the manuscript.
